# Supplementary material for: Evaluation of Spatial Distribution of Three Major Leptocorisa (Hemiptera: Alydidae) Pests Using MaxEnt Model
Source: Insects. 2022 Aug 20;13(8):750. doi: 10.3390/insects13080750 (PMC9409444; doi:10.3390/insects13080750)
Supplement: Supplementary file 1 [file insects-13-00750-s001.zip › Supplementary table S1.pdf]

Table S1. Pearson correlation matrix of bioclimatic variables in *L. chinensis*

		Bio1	Bio2	Bio3	Bio4	Bio5	Bio6	Bio7	Bio8	Bio9	Bio10	Bio11	Bio12	Bio13	Bio14	Bio15	Bio16	Bio17	Bio18	Bio19	Elevation
**Bio1**	**Pearson correlation**	1	-.700**	-.069	-.852**	.599**	.945**	-.825**	.605**	.927**	.909**	.977**	.084	-.250	.136	-.319*	-.165	.088	-.234	.108	-.598**
	**Sig. (2-tailed)**		.000	.628	.000	.000	.000	.000	.000	.000	.000	.000	.557	.076	.343	.023	.247	.541	.098	.451	.000
	**N**	51	51	51	51	51	51	51	51	51	51	51	51	51	51	51	51	51	51	51	51
**Bio2**	**Pearson correlation**	-.700**	1	.640**	.859**	-.039	-.859**	.934**	-.295*	-.857**	-.464**	-.792**	-.328*	.166	-.425**	.444**	.052	-.414**	.187	-.439**	.165
	**Sig. (2-tailed)**	.000		.000	.000	.788	.000	.000	.036	.000	.001	.000	.019	.244	.002	.001	.716	.003	.188	.001	.246
	**N**	51	51	51	51	51	51	51	51	51	51	51	51	51	51	51	51	51	51	51	51
**Bio3**	**Pearson correlation**	-.069	.640**	1	.175	.173	-.238	.325*	-.067	-.273	-.029	-.130	-.173	.099	-.243	.233	-.009	-.278*	.123	-.297*	.031
	**Sig. (2-tailed)**	.628	.000		.218	.224	.093	.020	.640	.053	.842	.364	.224	.488	.086	.100	.947	.048	.389	.034	.830
	**N**	51	51	51	51	51	51	51	51	51	51	51	51	51	51	51	51	51	51	51	51
**Bio4**	**Pearson correlation**	-.852**	.859**	.175	1	-.162	-.939**	.977**	-.314*	-.920**	-.570**	-.936**	-.330*	.158	-.387**	.440**	.076	-.351*	.171	-.370**	.226
	**Sig. (2-tailed)**	.000	.000	.218		.256	.000	.000	.025	.000	.000	.000	.018	.268	.005	.001	.597	.011	.231	.007	.110
	**N**	51	51	51	51	51	51	51	51	51	51	51	51	51	51	51	51	51	51	51	51
**Bio5**	**Pearson correlation**	.599**	-.039	.173	-.162	1	.435**	-.117	.370**	.379**	.856**	.483**	-.208	-.455**	-.011	-.292*	-.422**	-.041	-.401**	-.040	-.667**
	**Sig. (2-tailed)**	.000	.788	.224	.256		.001	.414	.008	.006	.000	.000	.144	.001	.937	.038	.002	.773	.004	.783	.000
	**N**	51	51	51	51	51	51	51	51	51	51	51	51	51	51	51	51	51	51	51	51
**Bio6**	**Pearson correlation**	.945**	-.859**	-.238	-.939**	.435**	1	-.945**	.421**	.968**	.781**	.986**	.235	-.283*	.371**	-.491**	-.191	.336*	-.280*	.357*	-.385**
	**Sig. (2-tailed)**	.000	.000	.093	.000	.001		.000	.002	.000	.000	.000	.097	.044	.007	.000	.180	.016	.047	.010	.005
	**N**	51	51	51	51	51	51	51	51	51	51	51	51	51	51	51	51	51	51	51	51
**Bio7**	**Pearson correlation**	-.825**	.934**	.325*	.977**	-.117	-.945**	1	-.330*	-.930**	-.550**	-.912**	-.335*	.147	-.414**	.436**	.057	-.386**	.163	-.408**	.183
	**Sig. (2-tailed)**	.000	.000	.020	.000	.414	.000		.018	.000	.000	.000	.016	.304	.003	.001	.689	.005	.252	.003	.200
	**N**	51	51	51	51	51	51	51	51	51	51	51	51	51	51	51	51	51	51	51	51
**Bio8**	**Pearson correlation**	.605**	-.295*	-.067	-.314*	.370**	.421**	-.330*	1	.514**	.636**	.475**	-.063	.227	-.400**	.368**	.317*	-.425**	.267	-.402**	-.691**
	**Sig. (2-tailed)**	.000	.036	.640	.025	.008	.002	.018		.000	.000	.000	.659	.110	.004	.008	.023	.002	.058	.003	.000
	**N**	51	51	51	51	51	51	51	51	51	51	51	51	51	51	51	51	51	51	51	51
**Bio9**	**Pearson correlation**	.927**	-.857**	-.273	-.920**	.379**	.968**	-.930**	.514**	1	.756**	.960**	.268	-.179	.313*	-.397**	-.078	.279*	-.170	.306*	-.386**
	**Sig. (2-tailed)**	.000	.000	.053	.000	.006	.000	.000	.000		.000	.000	.057	.210	.026	.004	.584	.047	.234	.029	.005
	**N**	51	51	51	51	51	51	51	51	51	51	51	51	51	51	51	51	51	51	51	51
**Bio10**	**Pearson correlation**	.909**	-.464**	-.029	-.570**	.856**	.781**	-.550**	.636**	.756**	1	.821**	-.104	-.353*	.006	-.269	-.280*	-.036	-.321*	-.021	-.754**
	**Sig. (2-tailed)**	.000	.001	.842	.000	.000	.000	.000	.000	.000		.000	.467	.011	.964	.056	.047	.801	.022	.884	.000
	**N**	51	51	51	51	51	51	51	51	51	51	51	51	51	51	51	51	51	51	51	51
**Bio11**	**Pearson correlation**	.977**	-.792**	-.130	-.936**	.483**	.986**	-.912**	.475**	.960**	.821**	1	.189	-.270	.285*	-.434**	-.182	.242	-.263	.262	-.465**
	**Sig. (2-tailed)**	.000	.000	.364	.000	.000	.000	.000	.000	.000	.000		.183	.056	.042	.001	.200	.087	.063	.063	.001
	**N**	51	51	51	51	51	51	51	51	51	51	51	51	51	51	51	51	51	51	51	51
**Bio12**	**Pearson correlation**	.084	-.328*	-.173	-.330*	-.208	.235	-.335*	-.063	.268	-.104	.189	1	.502**	.444**	-.164	.564**	.499**	.533**	.517**	.311*
	**Sig. (2-tailed)**	.557	.019	.224	.018	.144	.097	.016	.659	.057	.467	.183		.000	.001	.250	.000	.000	.000	.000	.026
	**N**	51	51	51	51	51	51	51	51	51	51	51	51	51	51	51	51	51	51	51	51
**Bio13**	**Pearson correlation**	-.250	.166	.099	.158	-.455**	-.283*	.147	.227	-.179	-.353*	-.270	.502**	1	-.446**	.751**	.976**	-.385**	.973**	-.369**	.209
	**Sig. (2-tailed)**	.076	.244	.488	.268	.001	.044	.304	.110	.210	.011	.056	.000		.001	.000	.000	.005	.000	.008	.141
	**N**	51	51	51	51	51	51	51	51	51	51	51	51	51	51	51	51	51	51	51	51
**Bio14**	**Pearson correlation**	.136	-.425**	-.243	-.387**	-.011	.371**	-.414**	-.400**	.313*	.006	.285*	.444**	-.446**	1	-.886**	-.406**	.991**	-.419**	.990**	.373**
	**Sig. (2-tailed)**	.343	.002	.086	.005	.937	.007	.003	.004	.026	.964	.042	.001	.001		.000	.003	.000	.002	.000	.007

	**N**	51	51	51	51	51	51	51	51	51	51	51	51	51	51	51	51	51	51	51	51
**Bio15**	**Pearson correlation**	-.319*	.444**	.233	.440**	-.292*	-.491**	.436**	.368**	-.397**	-.269	-.434**	-.164	.751**	-.886**	1	.707**	-.858**	.730**	-.853**	-.096
	**Sig. (2-tailed)**	.023	.001	.100	.001	.038	.000	.001	.008	.004	.056	.001	.250	.000	.000		.000	.000	.000	.000	.503
	**N**	51	51	51	51	51	51	51	51	51	51	51	51	51	51	51	51	51	51	51	51
**Bio16**	**Pearson correlation**	-.165	.052	-.009	.076	-.422**	-.191	.057	.317*	-.078	-.280*	-.182	.564**	.976**	-.406**	.707**	1	-.343*	.973**	-.324*	.108
	**Sig. (2-tailed)**	.247	.716	.947	.597	.002	.180	.689	.023	.584	.047	.200	.000	.000	.003	.000		.014	.000	.020	.449
	**N**	51	51	51	51	51	51	51	51	51	51	51	51	51	51	51	51	51	51	51	51
**Bio17**	**Pearson correlation**	.088	-.414**	-.278*	-.351*	-.041	.336*	-.386**	-.425**	.279*	-.036	.242	.499**	-.385**	.991**	-.858**	-.343*	1	-.368**	.999**	.389**
	**Sig. (2-tailed)**	.541	.003	.048	.011	.773	.016	.005	.002	.047	.801	.087	.000	.005	.000	.000	.014		.008	.000	.005
	**N**	51	51	51	51	51	51	51	51	51	51	51	51	51	51	51	51	51	51	51	51
**Bio18**	**Pearson correlation**	-.234	.187	.123	.171	-.401**	-.280*	.163	.267	-.170	-.321*	-.263	.533**	.973**	-.419**	.730**	.973**	-.368**	1	-.351*	.174
	**Sig. (2-tailed)**	.098	.188	.389	.231	.004	.047	.252	.058	.234	.022	.063	.000	.000	.002	.000	.000	.008		.012	.223
	**N**	51	51	51	51	51	51	51	51	51	51	51	51	51	51	51	51	51	51	51	51
**Bio19**	**Pearson correlation**	.108	-.439**	-.297*	-.370**	-.040	.357*	-.408**	-.402**	.306*	-.021	.262	.517**	-.369**	.990**	-.853**	-.324*	.999**	-.351*	1	.388**
	**Sig. (2-tailed)**	.451	.001	.034	.007	.783	.010	.003	.003	.029	.884	.063	.000	.008	.000	.000	.020	.000	.012		.005
	**N**	51	51	51	51	51	51	51	51	51	51	51	51	51	51	51	51	51	51	51	51
**Elevation**	**Pearson correlation**	-.598**	.165	.031	.226	-.667**	-.385**	.183	-.691**	-.386**	-.754**	-.465**	.311*	.209	.373**	-.096	.108	.389**	.174	.388**	1
	**Sig. (2-tailed)**	.000	.246	.830	.110	.000	.005	.200	.000	.005	.000	.001	.026	.141	.007	.503	.449	.005	.223	.005	
	**N**	51	51	51	51	51	51	51	51	51	51	51	51	51	51	51	51	51	51	51	51

**. Correlation is significant at the 0.01 level (2-tailed).
*. Correlation is significant at the 0.05 level (2-tailed).
