# Supplementary material for: Evaluation of Spatial Distribution of Three Major Leptocorisa (Hemiptera: Alydidae) Pests Using MaxEnt Model
Source: Insects. 2022 Aug 20;13(8):750. doi: 10.3390/insects13080750 (PMC9409444; doi:10.3390/insects13080750)
Supplement: Supplementary file 1 [file insects-13-00750-s001.zip › Supplementary table S2.pdf]

Table S2. Pearson correlation matrix of bioclimatic variables in *L. acuta*

		Bio1	Bio2	Bio3	Bio4	Bio5	Bio6	Bio7	Bio8	Bio9	Bio10	Bio11	Bio12	Bio13	Bio14	Bio15	Bio16	Bio17	Bio18	Bio19	Elevation
**Bio1**	**Pearson correlation**	1	.019	.540**	-.693**	.791**	.854**	-.445**	.776**	.800**	.907**	.961**	.092	.252	-.392**	.658**	.320*	-.387**	-.054	.000	-.340**
	**Sig. (2-tailed)**		.887	.000	.000	.000	.000	.000	.000	.000	.000	.000	.489	.054	.002	.000	.013	.002	.685	.998	.008
	**N**	59	59	59	59	59	59	59	59	59	59	59	59	59	59	59	59	59	59	59	59
**Bio2**	**Pearson correlation**	.019	1	.432**	-.019	.476**	-.357**	.679**	-.146	.070	.008	.006	-.577**	-.384**	-.401**	.134	-.467**	-.409**	-.561**	-.228	-.075
	**Sig. (2-tailed)**	.887		.001	.887	.000	.006	.000	.269	.597	.950	.963	.000	.003	.002	.312	.000	.001	.000	.082	.574
	**N**	59	59	59	59	59	59	59	59	59	59	59	59	59	59	59	59	59	59	59	59
**Bio3**	**Pearson correlation**	.540**	.432**	1	-.856**	.399**	.543**	-.346**	.090	.722**	.210	.699**	-.133	-.008	-.321*	.251	-.016	-.272*	-.382**	.095	-.125
	**Sig. (2-tailed)**	.000	.001		.000	.002	.000	.007	.499	.000	.110	.000	.314	.955	.013	.056	.902	.037	.003	.472	.345
	**N**	59	59	59	59	59	59	59	59	59	59	59	59	59	59	59	59	59	59	59	59
**Bio4**	**Pearson correlation**	-.693**	-.019	-.856**	1	-.325*	-.833**	.708**	-.245	-.832**	-.330*	-.865**	-.118	-.233	.251	-.382**	-.268*	.209	.169	-.140	.070
	**Sig. (2-tailed)**	.000	.887	.000		.012	.000	.000	.061	.000	.011	.000	.373	.076	.055	.003	.040	.112	.201	.291	.597
	**N**	59	59	59	59	59	59	59	59	59	59	59	59	59	59	59	59	59	59	59	59
**Bio5**	**Pearson correlation**	.791**	.476**	.399**	-.325*	1	.419**	.157	.580**	.611**	.856**	.674**	-.230	-.047	-.472**	.579**	-.015	-.503**	-.333*	-.103	-.374**
	**Sig. (2-tailed)**	.000	.000	.002	.012		.001	.236	.000	.000	.000	.000	.080	.722	.000	.000	.910	.000	.010	.438	.003
	**N**	59	59	59	59	59	59	59	59	59	59	59	59	59	59	59	59	59	59	59	59
**Bio6**	**Pearson correlation**	.854**	-.357**	.543**	-.833**	.419**	1	-.831**	.548**	.830**	.644**	.921**	.317*	.377**	-.168	.461**	.462**	-.142	.073	.201	-.207
	**Sig. (2-tailed)**	.000	.006	.000	.000	.001		.000	.000	.000	.000	.000	.014	.003	.204	.000	.000	.283	.584	.128	.116
	**N**	59	59	59	59	59	59	59	59	59	59	59	59	59	59	59	59	59	59	59	59
**Bio7**	**Pearson correlation**	-.445**	.679**	-.346**	.708**	.157	-.831**	1	-.240	-.529**	-.177	-.590**	-.485**	-.439**	-.106	-.147	-.511**	-.153	-.283*	-.281*	-.004
	**Sig. (2-tailed)**	.000	.000	.007	.000	.236	.000		.067	.000	.180	.000	.000	.001	.422	.266	.000	.247	.030	.031	.976
	**N**	59	59	59	59	59	59	59	59	59	59	59	59	59	59	59	59	59	59	59	59
**Bio8**	**Pearson correlation**	.776**	-.146	.090	-.245	.580**	.548**	-.240	1	.279*	.856**	.626**	.180	.258*	-.206	.544**	.326*	-.208	.254	-.126	-.264*
	**Sig. (2-tailed)**	.000	.269	.499	.061	.000	.000	.067		.033	.000	.000	.171	.049	.117	.000	.012	.114	.052	.343	.044
	**N**	59	59	59	59	59	59	59	59	59	59	59	59	59	59	59	59	59	59	59	59
**Bio9**	**Pearson correlation**	.800**	.070	.722**	-.832**	.611**	.830**	-.529**	.279*	1	.580**	.882**	.106	.279*	-.380**	.517**	.313*	-.351**	-.196	.186	-.239
	**Sig. (2-tailed)**	.000	.597	.000	.000	.000	.000	.000	.033		.000	.000	.425	.032	.003	.000	.016	.006	.136	.158	.069
	**N**	59	59	59	59	59	59	59	59	59	59	59	59	59	59	59	59	59	59	59	59
**Bio10**	**Pearson correlation**	.907**	.008	.210	-.330*	.856**	.644**	-.177	.856**	.580**	1	.759**	.055	.188	-.351**	.621**	.259*	-.372**	-.001	-.038	-.427**
	**Sig. (2-tailed)**	.000	.950	.110	.011	.000	.000	.180	.000	.000		.000	.680	.154	.006	.000	.048	.004	.997	.775	.001
	**N**	59	59	59	59	59	59	59	59	59	59	59	59	59	59	59	59	59	59	59	59
**Bio11**	**Pearson correlation**	.961**	.006	.699**	-.865**	.674**	.921**	-.590**	.626**	.882**	.759**	1	.118	.265*	-.356**	.591**	.328*	-.337**	-.108	.078	-.271*
	**Sig. (2-tailed)**	.000	.963	.000	.000	.000	.000	.000	.000	.000	.000		.373	.043	.006	.000	.011	.009	.416	.555	.038
	**N**	59	59	59	59	59	59	59	59	59	59	59	59	59	59	59	59	59	59	59	59
**Bio12**	**Pearson correlation**	.092	-.577**	-.133	-.118	-.230	.317*	-.485**	.180	.106	.055	.118	1	.829**	.538**	-.042	.870**	.573**	.744**	.545**	.163
	**Sig. (2-tailed)**	.489	.000	.314	.373	.080	.014	.000	.171	.425	.680	.373		.000	.000	.750	.000	.000	.000	.000	.218
	**N**	59	59	59	59	59	59	59	59	59	59	59	59	59	59	59	59	59	59	59	59
**Bio13**	**Pearson correlation**	.252	-.384**	-.008	-.233	-.047	.377**	-.439**	.258*	.279*	.188	.265*	.829**	1	.082	.420**	.973**	.136	.735**	.409**	.170
	**Sig. (2-tailed)**	.054	.003	.955	.076	.722	.003	.001	.049	.032	.154	.043	.000		.537	.001	.000	.305	.000	.001	.199
	**N**	59	59	59	59	59	59	59	59	59	59	59	59	59	59	59	59	59	59	59	59
**Bio14**	**Pearson correlation**	-.392**	-.401**	-.321*	.251	-.472**	-.168	-.106	-.206	-.380**	-.351**	-.356**	.538**	.082	1	-.731**	.099	.979**	.280*	.399**	.101
	**Sig. (2-tailed)**	.002	.002	.013	.055	.000	.204	.422	.117	.003	.006	.006	.000	.537		.000	.457	.000	.032	.002	.448

	**N**	59	59	59	59	59	59	59	59	59	59	59	59	59	59	59	59	59	59	59	59
**Bio15**	**Pearson correlation**	.658**	.134	.251	-.382**	.579**	.461**	-.147	.544**	.517**	.621**	.591**	-.042	.420**	-.731**	1	.417**	-.733**	.166	-.253	.034
	**Sig. (2-tailed)**	.000	.312	.056	.003	.000	.000	.266	.000	.000	.000	.000	.750	.001	.000		.001	.000	.208	.053	.801
	**N**	59	59	59	59	59	59	59	59	59	59	59	59	59	59	59	59	59	59	59	59
**Bio16**	**Pearson correlation**	.320*	-.467**	-.016	-.268*	-.015	.462**	-.511**	.326*	.313*	.259*	.328*	.870**	.973**	.099	.417**	1	.138	.757**	.398**	.143
	**Sig. (2-tailed)**	.013	.000	.902	.040	.910	.000	.000	.012	.016	.048	.011	.000	.000	.457	.001		.298	.000	.002	.279
	**N**	59	59	59	59	59	59	59	59	59	59	59	59	59	59	59	59	59	59	59	59
**Bio17**	**Pearson correlation**	-.387**	-.409**	-.272*	.209	-.503**	-.142	-.153	-.208	-.351**	-.372**	-.337**	.573**	.136	.979**	-.733**	.138	1	.312*	.422**	.115
	**Sig. (2-tailed)**	.002	.001	.037	.112	.000	.283	.247	.114	.006	.004	.009	.000	.305	.000	.000	.298		.016	.001	.385
	**N**	59	59	59	59	59	59	59	59	59	59	59	59	59	59	59	59	59	59	59	59
**Bio18**	**Pearson correlation**	-.054	-.561**	-.382**	.169	-.333*	.073	-.283*	.254	-.196	-.001	-.108	.744**	.735**	.280*	.166	.757**	.312*	1	.043	.255
	**Sig. (2-tailed)**	.685	.000	.003	.201	.010	.584	.030	.052	.136	.997	.416	.000	.000	.032	.208	.000	.016		.746	.051
	**N**	59	59	59	59	59	59	59	59	59	59	59	59	59	59	59	59	59	59	59	59
**Bio19**	**Pearson correlation**	.000	-.228	.095	-.140	-.103	.201	-.281*	-.126	.186	-.038	.078	.545**	.409**	.399**	-.253	.398**	.422**	.043	1	-.004
	**Sig. (2-tailed)**	.998	.082	.472	.291	.438	.128	.031	.343	.158	.775	.555	.000	.001	.002	.053	.002	.001	.746		.973
	**N**	59	59	59	59	59	59	59	59	59	59	59	59	59	59	59	59	59	59	59	59
**Elevation**	**Pearson correlation**	-.340**	-.075	-.125	.070	-.374**	-.207	-.004	-.264*	-.239	-.427**	-.271*	.163	.170	.101	.034	.143	.115	.255	-.004	1
	**Sig. (2-tailed)**	.008	.574	.345	.597	.003	.116	.976	.044	.069	.001	.038	.218	.199	.448	.801	.279	.385	.051	.973	
	**N**	59	59	59	59	59	59	59	59	59	59	59	59	59	59	59	59	59	59	59	59

**. Correlation is significant at the 0.01 level (2-tailed).
*. Correlation is significant at the 0.05 level (2-tailed).
