# Supplementary material for: Evaluation of Spatial Distribution of Three Major Leptocorisa (Hemiptera: Alydidae) Pests Using MaxEnt Model
Source: Insects. 2022 Aug 20;13(8):750. doi: 10.3390/insects13080750 (PMC9409444; doi:10.3390/insects13080750)
Supplement: Supplementary file 1 [file insects-13-00750-s001.zip › Supplementary table S3.pdf]

Table S3. Pearson correlation matrix of bioclimatic variables in *L. oratoria*

		Bio1	Bio2	Bio3	Bio4	Bio5	Bio6	Bio7	Bio8	Bio9	Bio10	Bio11	Bio12	Bio13	Bio14	Bio15	Bio16	Bio17	Bio18	Bio19	Elevation
Bio1	Pearson correlation	1	.009	.062	-.271*	.885**	.802**	-.097	.890**	.924**	.929**	.917**	-.283*	-.210	-.122	.044	-.256	-.127	-.607**	.297*	-.886**
	Sig. (2-tailed)		.951	.661	.050	.000	.000	.487	.000	.000	.000	.000	.040	.132	.385	.756	.064	.363	.000	.031	.000
	N	53	53	53	53	53	53	53	53	53	53	53	53	53	53	53	53	53	53	53	53
Bio2	Pearson correlation	.009	1	-.193	.484**	.370**	-.415**	.759**	.246	-.178	.193	-.189	-.244	-.167	-.097	.173	-.171	-.154	-.201	-.061	-.121
	Sig. (2-tailed)	.951		.167	.000	.006	.002	.000	.076	.203	.167	.176	.078	.232	.488	.216	.222	.272	.150	.665	.390
	N	53	53	53	53	53	53	53	53	53	53	53	53	53	53	53	53	53	53	53	53
Bio3	Pearson correlation	.062	-.193	1	-.869**	-.278*	.499**	-.769**	-.269	.339*	-.272*	.404**	.390**	-.087	.455**	-.579**	-.016	.475**	-.025	.228	.062
	Sig. (2-tailed)	.661	.167		.000	.044	.000	.000	.051	.013	.049	.003	.004	.538	.001	.000	.911	.000	.859	.101	.658
	N	53	53	53	53	53	53	53	53	53	53	53	53	53	53	53	53	53	53	53	53
Bio4	Pearson correlation	-.271*	.484**	-.869**	1	.138	-.748**	.915**	.152	-.580**	.095	-.630**	-.268	.114	-.272*	.456**	.057	-.298*	.185	-.213	.037
	Sig. (2-tailed)	.050	.000	.000		.325	.000	.000	.278	.000	.500	.000	.052	.417	.048	.001	.683	.030	.185	.125	.790
	N	53	53	53	53	53	53	53	53	53	53	53	53	53	53	53	53	53	53	53	53
Bio5	Pearson correlation	.885**	.370**	-.278*	.138	1	.475**	.350*	.941**	.699**	.975**	.661**	-.447**	-.224	-.251	.242	-.294*	-.281*	-.626**	.227	-.844**
	Sig. (2-tailed)	.000	.006	.044	.325		.000	.010	.000	.000	.000	.000	.001	.107	.070	.081	.033	.042	.000	.103	.000
	N	53	53	53	53	53	53	53	53	53	53	53	53	53	53	53	53	53	53	53	53
Bio6	Pearson correlation	.802**	-.415**	.499**	-.748**	.475**	1	-.658**	.482**	.947**	.556**	.958**	-.033	-.171	.082	-.229	-.185	.101	-.477**	.364**	-.597**
	Sig. (2-tailed)	.000	.002	.000	.000	.000		.000	.000	.000	.000	.000	.813	.222	.561	.099	.185	.471	.000	.007	.000
	N	53	53	53	53	53	53	53	53	53	53	53	53	53	53	53	53	53	53	53	53
Bio7	Pearson correlation	-.097	.759**	-.769**	.915**	.350*	-.658**	1	.292*	-.410**	.242	-.454**	-.347*	-.010	-.302*	.450**	-.054	-.348*	-.028	-.194	-.087
	Sig. (2-tailed)	.487	.000	.000	.000	.010	.000		.034	.002	.080	.001	.011	.944	.028	.001	.699	.011	.843	.164	.537
	N	53	53	53	53	53	53	53	53	53	53	53	53	53	53	53	53	53	53	53	53
Bio8	Pearson correlation	.890**	.246	-.269	.152	.941**	.482**	.292*	1	.659**	.966**	.650**	-.455**	-.267	-.231	.208	-.323*	-.250	-.516**	.111	-.906**
	Sig. (2-tailed)	.000	.076	.051	.278	.000	.000	.034		.000	.000	.000	.001	.054	.096	.134	.018	.071	.000	.430	.000
	N	53	53	53	53	53	53	53	53	53	53	53	53	53	53	53	53	53	53	53	53
Bio9	Pearson correlation	.924**	-.178	.339*	-.580**	.699**	.947**	-.410**	.659**	1	.747**	.989**	-.116	-.172	.015	-.143	-.206	.019	-.588**	.418**	-.743**
	Sig. (2-tailed)	.000	.203	.013	.000	.000	.000	.002	.000		.000	.000	.408	.219	.917	.308	.138	.891	.000	.002	.000
	N	53	53	53	53	53	53	53	53	53	53	53	53	53	53	53	53	53	53	53	53
Bio10	Pearson correlation	.929**	.193	-.272*	.095	.975**	.556**	.242	.966**	.747**	1	.712**	-.407**	-.182	-.234	.216	-.257	-.251	-.582**	.251	-.896**
	Sig. (2-tailed)	.000	.167	.049	.500	.000	.000	.080	.000	.000		.000	.003	.192	.092	.121	.063	.070	.000	.069	.000
	N	53	53	53	53	53	53	53	53	53	53	53	53	53	53	53	53	53	53	53	53
Bio11	Pearson correlation	.917**	-.189	.404**	-.630**	.661**	.958**	-.454**	.650**	.989**	.712**	1	-.123	-.217	.005	-.149	-.233	.010	-.581**	.340*	-.729**
	Sig. (2-tailed)	.000	.176	.003	.000	.000	.000	.001	.000	.000	.000		.378	.119	.971	.286	.093	.942	.000	.013	.000
	N	53	53	53	53	53	53	53	53	53	53	53	53	53	53	53	53	53	53	53	53
Bio12	Pearson correlation	-.283*	-.244	.390**	-.268	-.447**	-.033	-.347*	-.455**	-.116	-.407**	-.123	1	.736**	.686**	-.453**	.765**	.704**	.640**	.478**	.326*
	Sig. (2-tailed)	.040	.078	.004	.052	.001	.813	.011	.001	.408	.003	.378		.000	.000	.001	.000	.000	.000	.000	.017
	N	53	53	53	53	53	53	53	53	53	53	53	53	53	53	53	53	53	53	53	53
Bio13	Pearson correlation	-.210	-.167	-.087	.114	-.224	-.171	-.010	-.267	-.172	-.182	-.217	.736**	1	.161	.175	.979**	.177	.480**	.429**	.226
	Sig. (2-tailed)	.132	.232	.538	.417	.107	.222	.944	.054	.219	.192	.119	.000		.249	.210	.000	.206	.000	.001	.104
	N	53	53	53	53	53	53	53	53	53	53	53	53	53	53	53	53	53	53	53	53
Bio14	Pearson correlation	-.122	-.097	.455**	-.272*	-.251	.082	-.302*	-.231	.015	-.234	.005	.686**	.161	1	-.796**	.163	.987**	.450**	.452**	.125
	Sig. (2-tailed)	.385	.488	.001	.048	.070	.561	.028	.096	.917	.092	.971	.000	.249		.000	.243	.000	.001	.001	.373

	N	53	53	53	53	53	53	53	53	53	53	53	53	53	53	53	53	53	53	53	53
	Pearson correlation	.044	.173	-.579**	.456**	.242	-.229	.450**	.208	-.143	.216	-.149	-.453**	.175	-.796**	1	.180	-.826**	-.270	-.283*	-.023
Bio15	Sig. (2-tailed)	.756	.216	.000	.001	.081	.099	.001	.134	.308	.121	.286	.001	.210	.000		.196	.000	.050	.040	.868
	N	53	53	53	53	53	53	53	53	53	53	53	53	53	53	53	53	53	53	53	53
	Pearson correlation	-.256	-.171	-.016	.057	-.294*	-.185	-.054	-.323*	-.206	-.257	-.233	.765**	.979**	.163	.180	1	.169	.502**	.365**	.279*
Bio16	Sig. (2-tailed)	.064	.222	.911	.683	.033	.185	.699	.018	.138	.063	.093	.000	.000	.243	.196		.227	.000	.007	.043
	N	53	53	53	53	53	53	53	53	53	53	53	53	53	53	53	53	53	53	53	53
	Pearson correlation	-.127	-.154	.475**	-.298*	-.281*	.101	-.348*	-.250	.019	-.251	.010	.704**	.177	.987**	-.826**	.169	1	.482**	.452**	.131
Bio17	Sig. (2-tailed)	.363	.272	.000	.030	.042	.471	.011	.071	.891	.070	.942	.000	.206	.000	.000	.227		.000	.001	.350
	N	53	53	53	53	53	53	53	53	53	53	53	53	53	53	53	53	53	53	53	53
	Pearson correlation	-.607**	-.201	-.025	.185	-.626**	-.477**	-.028	-.516**	-.588**	-.582**	-.581**	.640**	.480**	.450**	-.270	.502**	.482**	1	-.032	.546**
Bio18	Sig. (2-tailed)	.000	.150	.859	.185	.000	.000	.843	.000	.000	.000	.000	.000	.000	.001	.050	.000	.000		.822	.000
	N	53	53	53	53	53	53	53	53	53	53	53	53	53	53	53	53	53	53	53	53
	Pearson correlation	.297*	-.061	.228	-.213	.227	.364**	-.194	.111	.418**	.251	.340*	.478**	.429**	.452**	-.283*	.365**	.452**	-.032	1	-.253
Bio19	Sig. (2-tailed)	.031	.665	.101	.125	.103	.007	.164	.430	.002	.069	.013	.000	.001	.001	.040	.007	.001	.822		.068
	N	53	53	53	53	53	53	53	53	53	53	53	53	53	53	53	53	53	53	53	53
	Pearson correlation	-.886**	-.121	.062	.037	-.844**	-.597**	-.087	-.906**	-.743**	-.896**	-.729**	.326*	.226	.125	-.023	.279*	.131	.546**	-.253	1
Elevation	Sig. (2-tailed)	.000	.390	.658	.790	.000	.000	.537	.000	.000	.000	.000	.017	.104	.373	.868	.043	.350	.000	.068	
	N	53	53	53	53	53	53	53	53	53	53	53	53	53	53	53	53	53	53	53	53

**. Correlation is significant at the 0.01 level (2-tailed).

*. Correlation is significant at the 0.05 level (2-tailed).
