# Supplementary figures and images for: Evaluation of Spatial Distribution of Three Major Leptocorisa (Hemiptera: Alydidae) Pests Using MaxEnt Model
Source: Insects. 2022 Aug 20;13(8):750. doi: 10.3390/insects13080750 (PMC9409444; doi:10.3390/insects13080750)

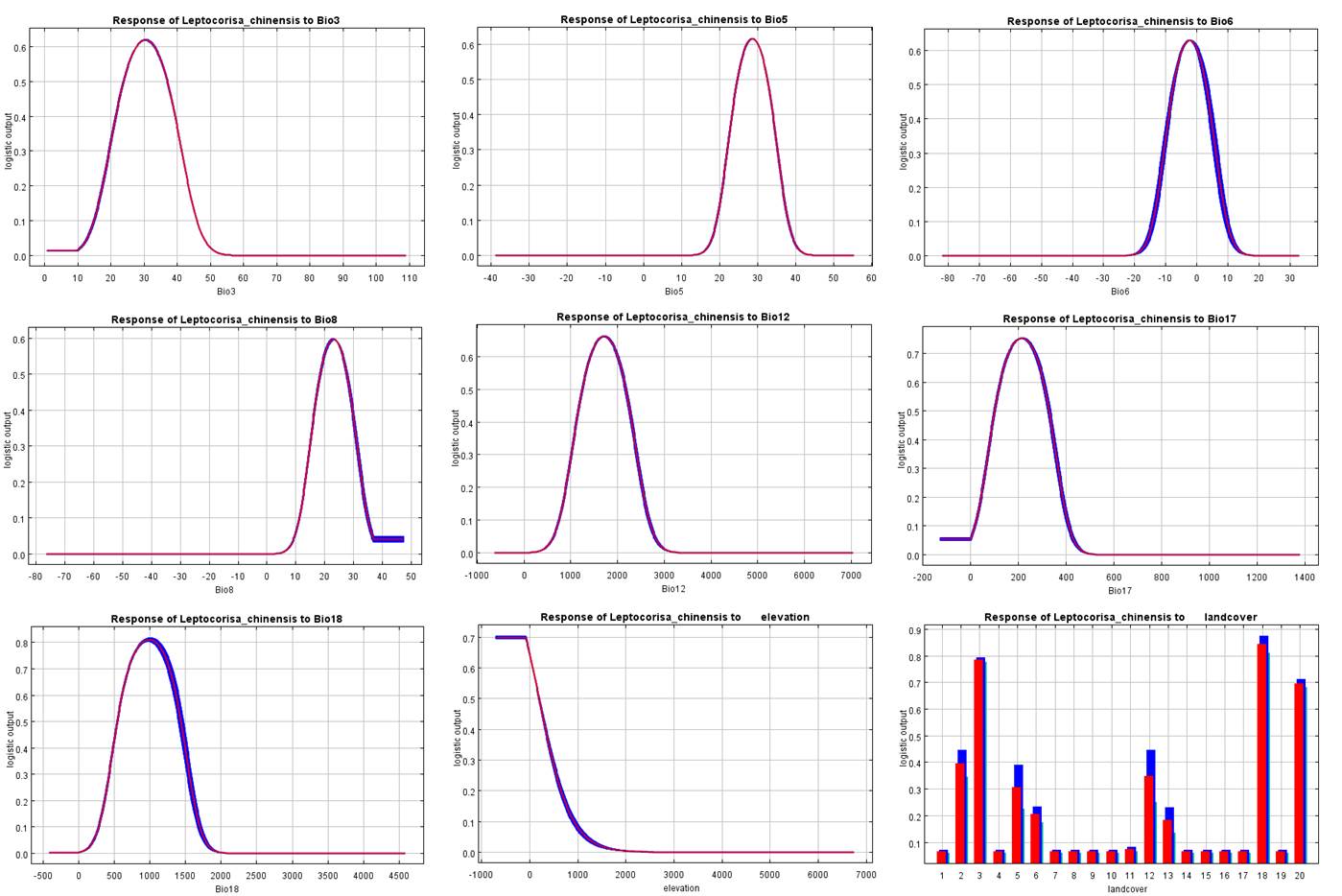

Supplement: Supplementary file 1 [file insects-13-00750-s001.zip › Supplementary Figure S1.jpg]

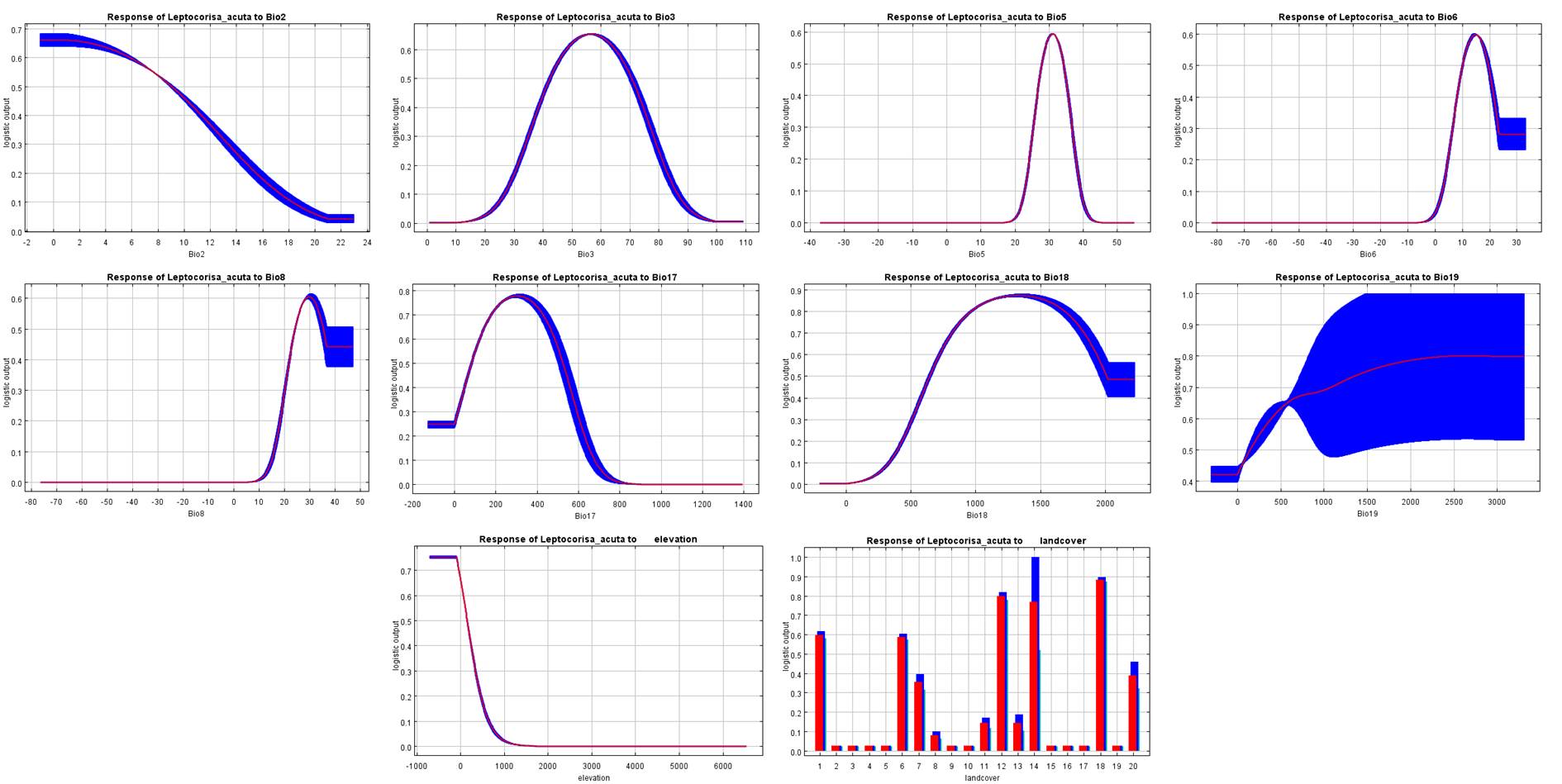

Supplement: Supplementary file 1 [file insects-13-00750-s001.zip › Supplementary Figure S2.jpg]

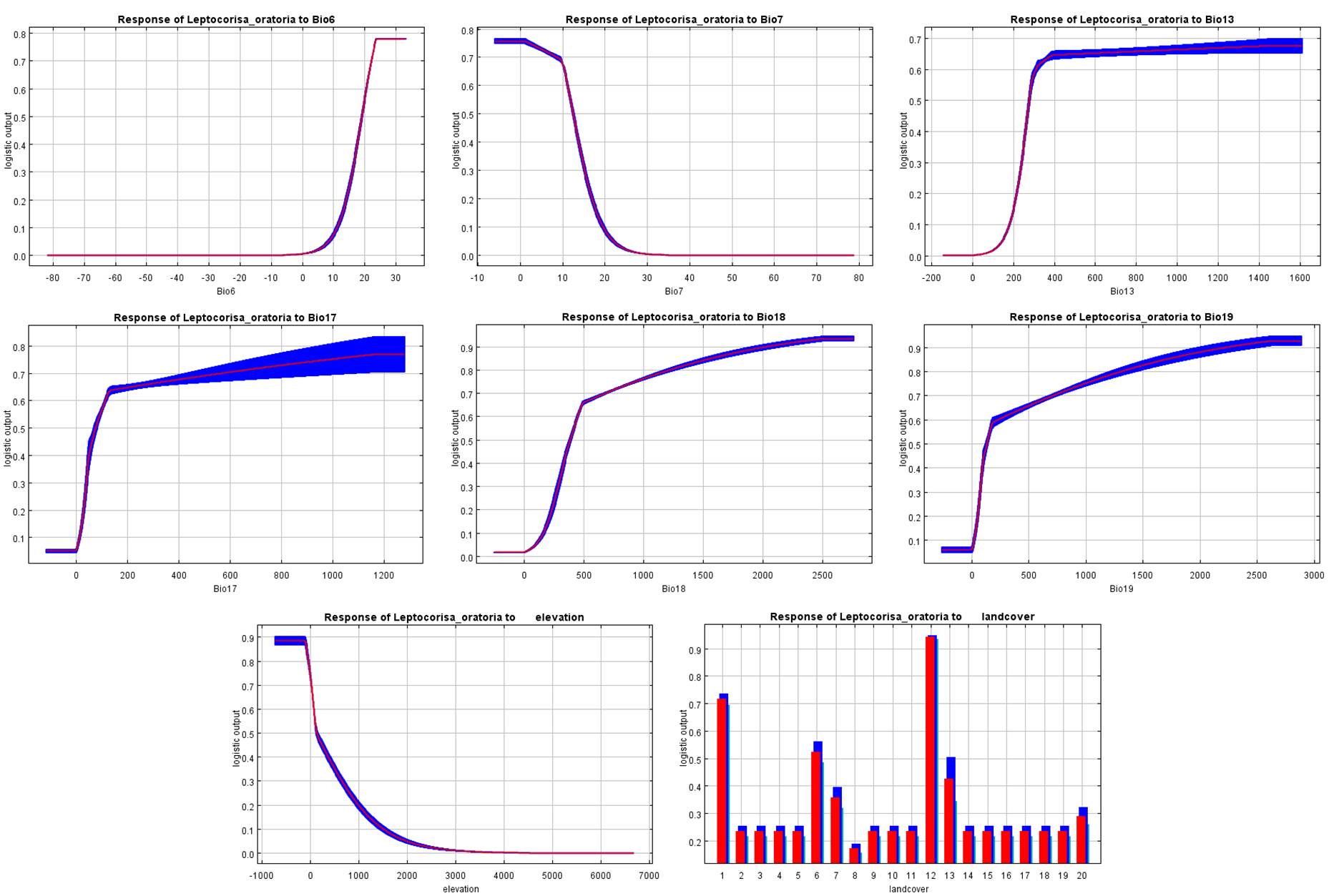

Supplement: Supplementary file 1 [file insects-13-00750-s001.zip › Supplementary Figure S3.jpg]

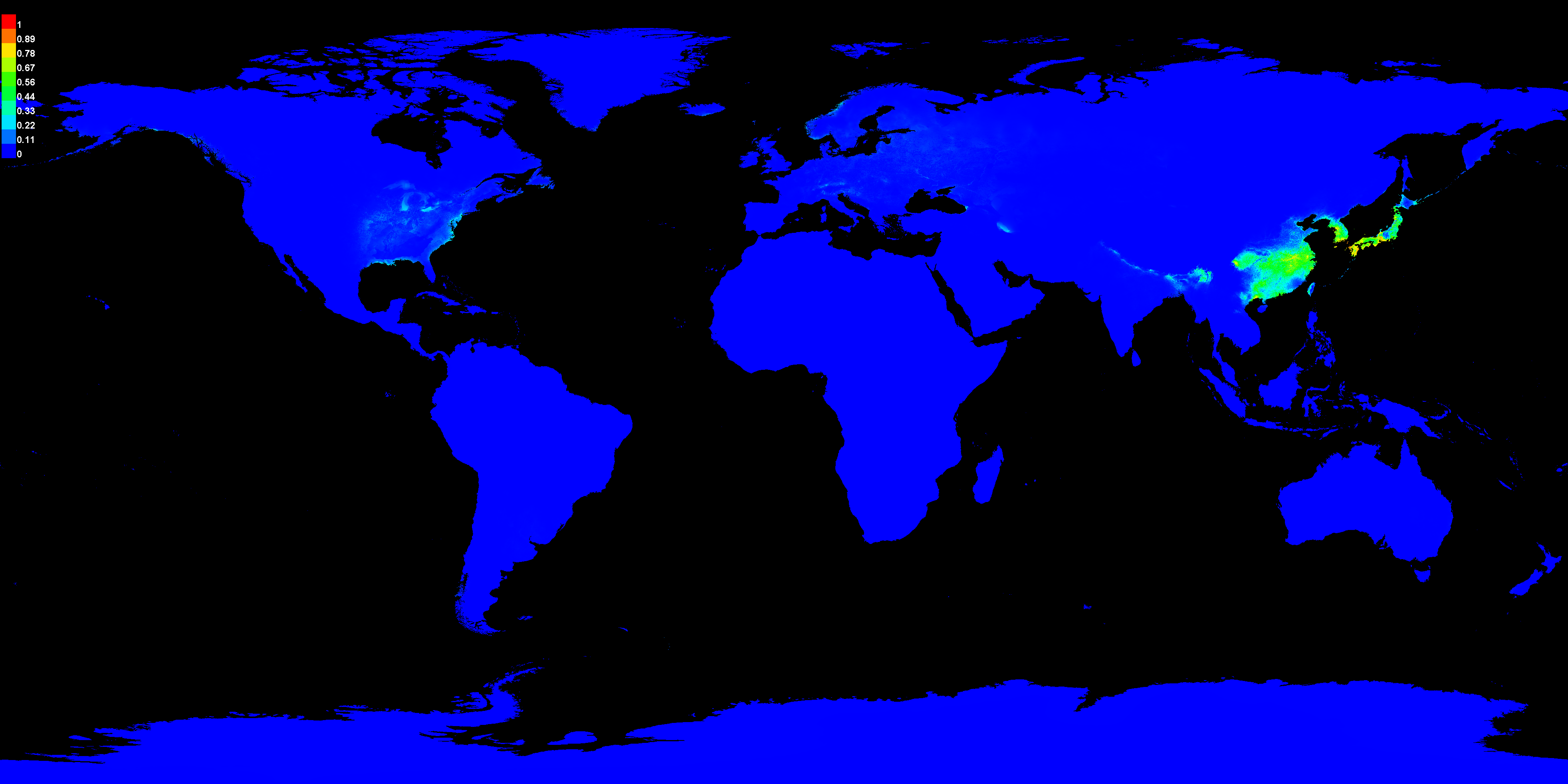

Supplement: Supplementary file 1 [file insects-13-00750-s001.zip › Supplementary Figure S4.jpg]

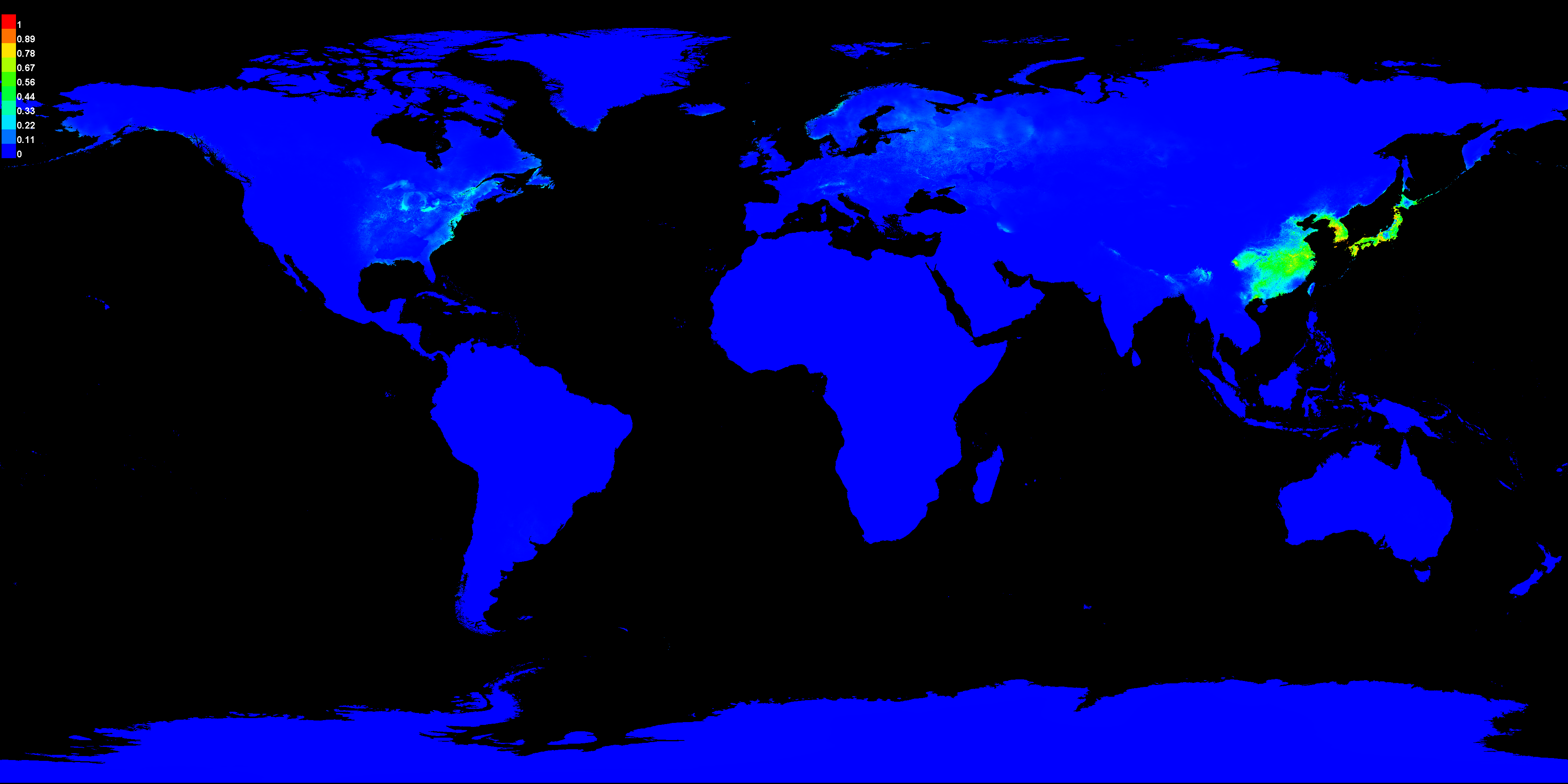

Supplement: Supplementary file 1 [file insects-13-00750-s001.zip › Supplementary Figure S5.jpg]

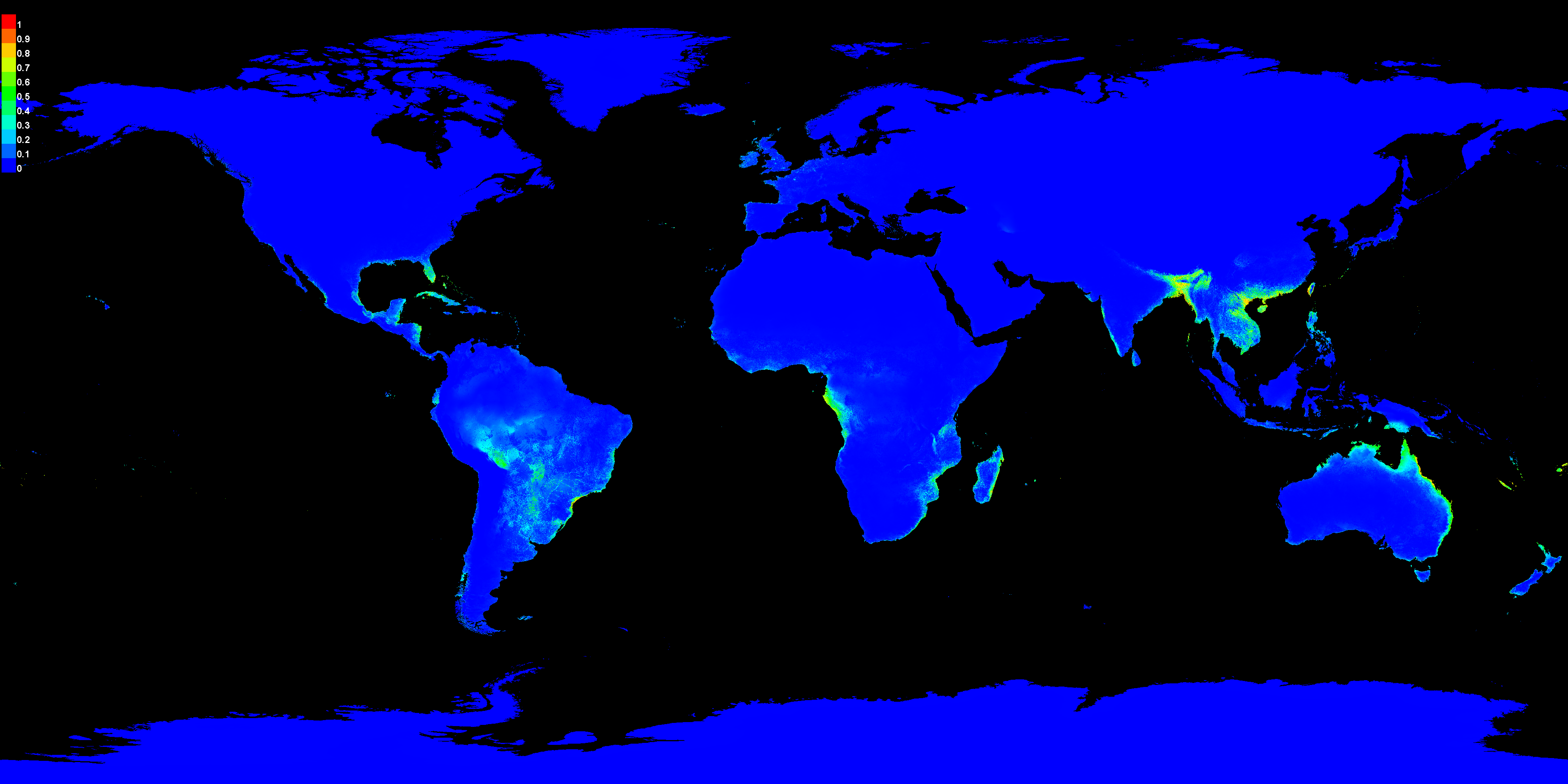

Supplement: Supplementary file 1 [file insects-13-00750-s001.zip › Supplementary Figure S6.jpg]

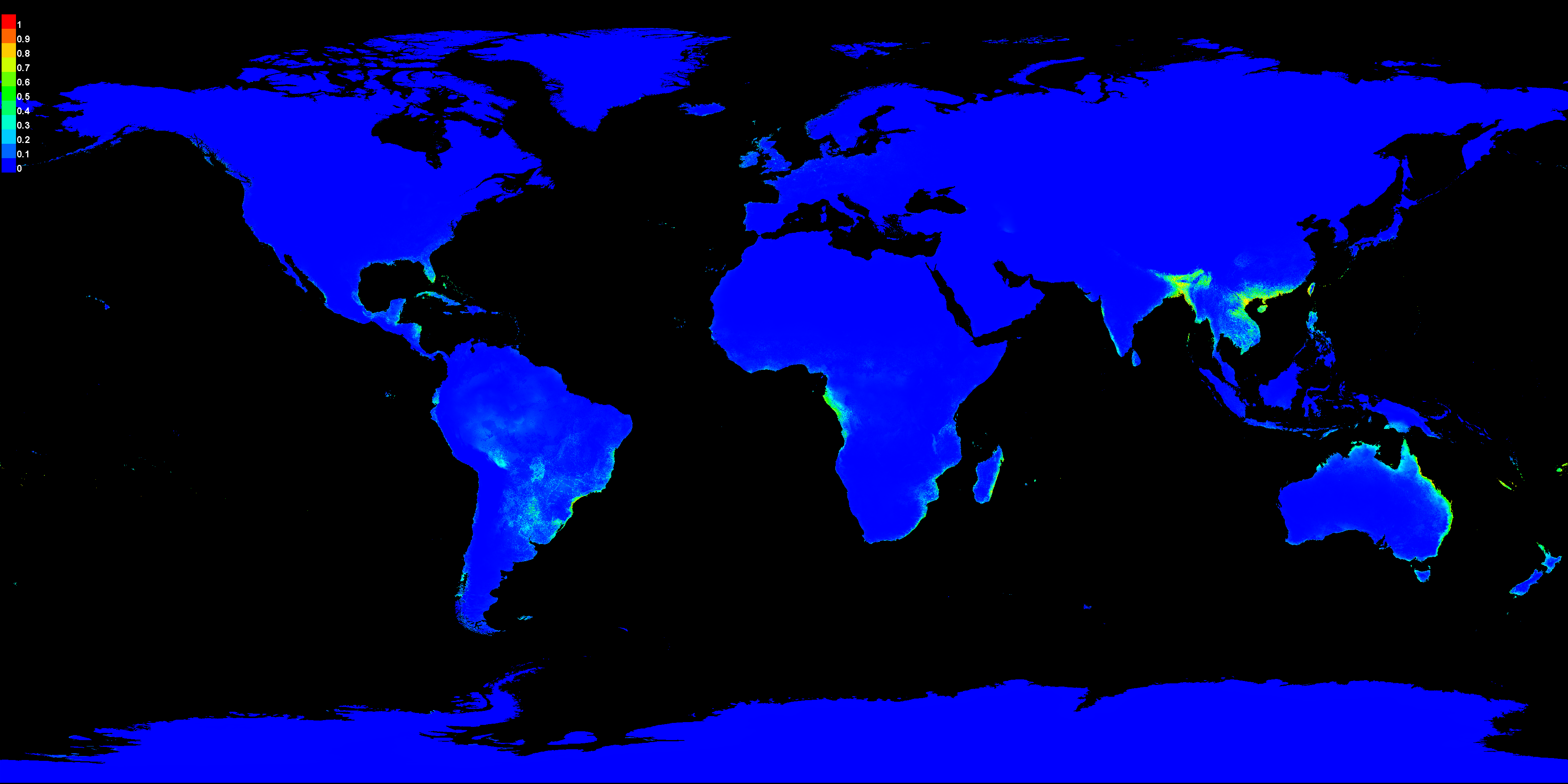

Supplement: Supplementary file 1 [file insects-13-00750-s001.zip › Supplementary Figure S7.jpg]

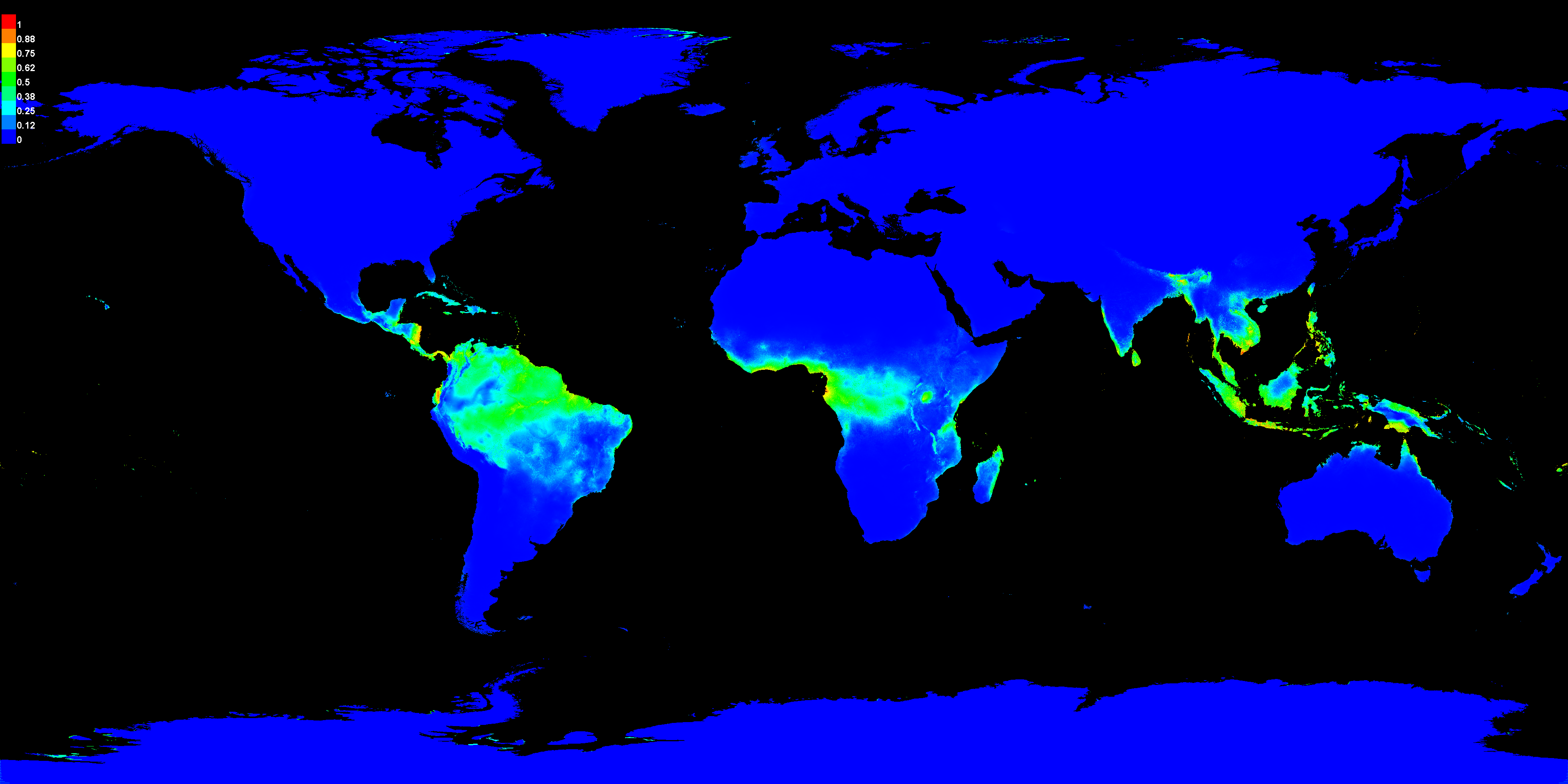

Supplement: Supplementary file 1 [file insects-13-00750-s001.zip › Supplementary Figure S8.jpg]

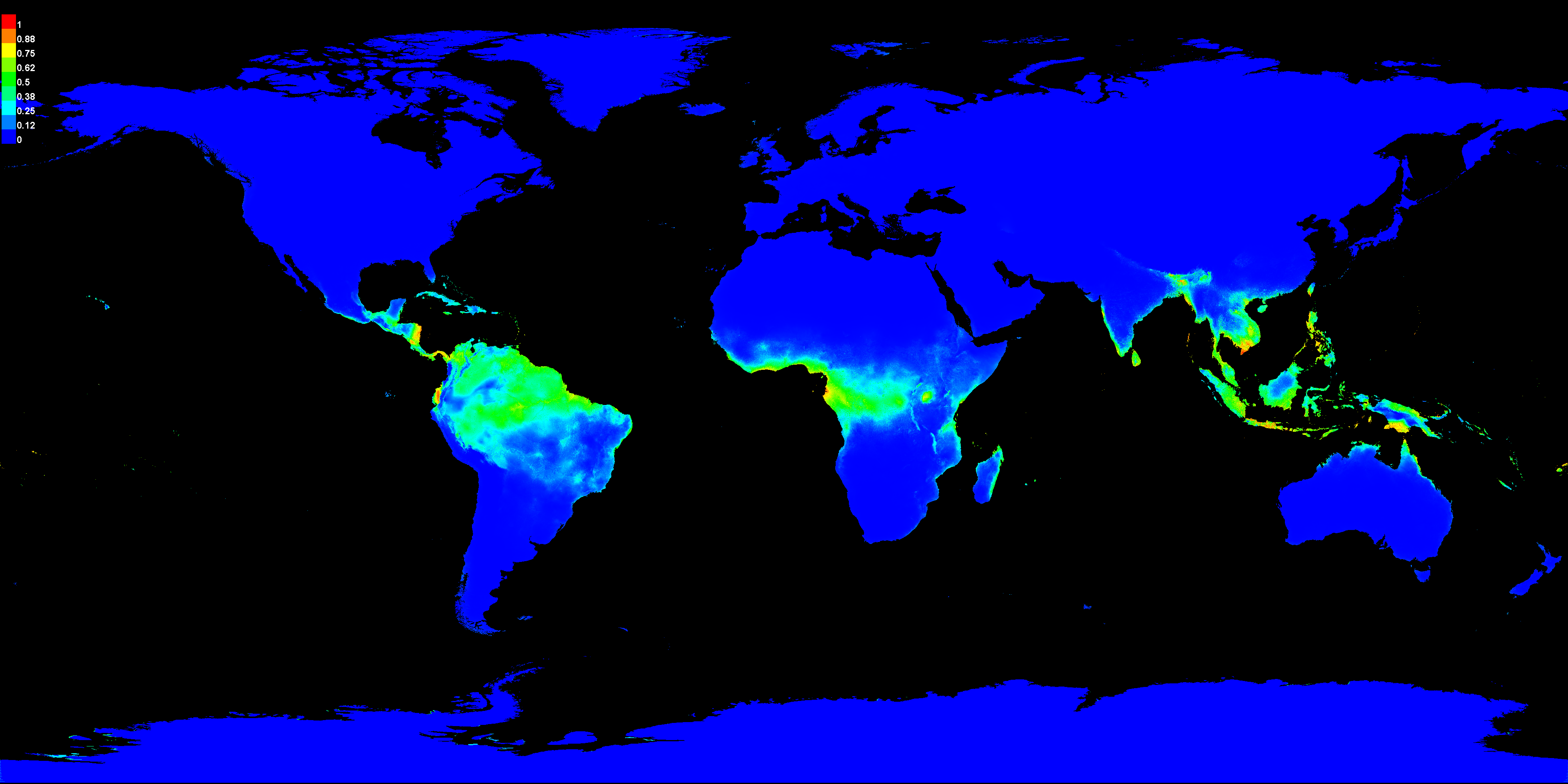

Supplement: Supplementary file 1 [file insects-13-00750-s001.zip › Supplementary Figure S9.jpg]
